# Constitutive androstane receptor agonist CITCO inhibits growth and expansion of brain tumour stem cells

**DOI:** 10.1038/sj.bjc.6606064

**Published:** 2011-01-11

**Authors:** S Chakraborty, S Kanakasabai, J J Bright

**Affiliations:** 1 Neuroscience Research Laboratory, Methodist Research Institute, 1800 N. Capitol Avenue, Noyes Bldg E504C, Indianapolis, IN 46202, USA; 2 Department of Medicine, Indiana University School of Medicine, Indianapolis, IN 46202, USA

**Keywords:** brain tumour stem cell, nuclear receptor, anticancer agent, cancer chemotherapy

## Abstract

**Background:**

Brain tumours present unique challenges to conventional therapies and pose major health problems around the world. Brain tumour stem cells (BTSCs) represent a small fraction of tumour cells that maintain growth, drug resistance and recurrence properties. Constitutive androstane receptor (CAR) is a nuclear receptor transcription factor that regulates drug metabolism and homoeostasis. In this study, we examined the effect of CAR agonist, 6-(4-chlorophenyl)imidazo[2,1-b][1,3]thiazole-5-carbaldehydeO-(3,4-dichlorobenzyl)oxime (CITCO) on BTSCs.

**Methods:**

The expression of CAR in BTSCs was detected by quantitative RT–PCR and western blot. The antiproliferative effect of CITCO on BTSCs was determined by WST-1 and ^3^H thymidine uptake assays. The effect of CITCO on CD133 expression, cell cycle progression and apoptosis in BTSCs was analysed by immunostaining and flow cytometry. The *in vivo* effect of CITCO was studied using subcutaneous (s.c.) BTSC xenograft in nude mice.

**Results:**

We show for the first time that BTSCs express altered levels of nuclear receptors compared with glioma cells. The expression of CAR mRNA and protein was low in BTSCs and that increased following treatment with CITCO in culture. CITCO induced a dose-dependent decrease in growth and expansion of CD133^+^ BTSCs as gliospheres in culture. Cell cycle arrest and apoptosis in BTSCs were induced by CITCO, but not in normal astrocytes. Growth of s.c BTSC xenograft in nude mice was also inhibited by CITCO.

**Conclusion:**

These findings indicate that CITCO inhibits the growth and expansion of BTSCs, suggesting the use of CAR agonists for the treatment of brain tumour.

Brain tumours are among the most devastating cancers that present unique challenges to therapy and pose major health problems around the world. Among many different types of tumours, glioblastoma is the most frequent primary malignant brain tumour in adults. Standard therapy includes surgical resection to the extent that is safely feasible, followed by radiation and chemotherapy, which have significant side effects and limited efficacy (Deorah *et al*, 2006), (Peacock and Lesser, 2006). Despite recent advances in surgery, radiation and chemotherapy, median survival is less than 1 year and a cure for brain tumour remains elusive. Multidrug resistance and fast recurrence are some of the challenges in combating brain tumours. Cancer stem cells (CSCs) are a small population of cells in cancer tissues with asymmetric division, self-renewal and tumour initiation capabilities. In acute myeloid leukaemia, CSCs were first identified (Bonnet and Dick, 1997) and subsequently in breast (Al-Hajj *et al*, 2003), prostate (Patrawala *et al*, 2006), liver (Yang *et al*, 2008), colon (Dalerba *et al*, 2007; O’Brien *et al*, 2007), pancreas (Li *et al*, 2007) and skin cancers (Schatton *et al*, 2008). Brain tumour stem cells (BTSCs) have also been isolated from gliomas that are positive for CD133 and posses tumour initiation potential in NOD/SCID mice (Singh *et al*, 2004). The BTSCs are resistant to standard therapies and are considered responsible for the recurrence of brain tumours after radiation and chemotherapy in patients (Singh *et al*, 2004; Bao *et al*, 2006). The failure to cure brain tumour has been attributed to the fact that typical therapies target rapidly proliferating tumour cells, which respond transiently, while sparing the highly tumourigenic BTSCs (Bao *et al*, 2006; Stupp and Hegi, 2007).

Nuclear hormone receptors are a family of transcriptional factors that regulate cell growth, differentiation and homoeostasis. Genomic studies have cloned 48 and 50 nuclear receptors in human and rodents, respectively, and many of them have therapeutic values in human diseases (Blumberg and Evans, 1998). The effect of nuclear receptor agonists on brain tumours has been shown recently (Naveilhan *et al*, 1994; Grommes *et al*, 2006; Papi *et al*, 2009), but their use in targeting BTSCs is not known. Constitutive androstane receptor (CAR, NR1I3) is an orphan nuclear receptor that contains a DNA-binding domain but uniquely lacking an activation domain (Baes *et al*, 1994). The CAR is retained in the cytoplasm by forming a complex with phosphatase 2A, HSP90 and cytosolic CAR retention protein (Kobayashi *et al*, 2003). Phenobarbital, 5*β*-pregnane-3,20-dione, and 5-androstan-3-ol are known endogenous CAR ligands (Moore *et al*, 2000). The hepatomitogen 1,4-Bis[2-(3,5-dichloropyridyloxy)]benzene (TCPOBOP) is a synthetic agonist for murine CAR (Tzameli *et al*, 2000) and 6-(4-chlorophenyl)imidazo[2,1-b][1,3]thiazole-5-carbaldehydeO-(3,4-dichlorobenzyl)oxime (CITCO) is an imidazothiazole derivative that functions as a selective agonist for human CAR (Maglich *et al*, 2003). Upon activation with specific agonist, CAR translocates into the nucleus and binds to the response elements as monomers or CAR/RXR heterodimers (Baes *et al*, 1994). The CAR functions as a xenobiotic receptor that regulates detoxification and clearance of toxic substances from the liver (Qatanani and Moore, 2005); however, its role in cancer is not known.

In this study, we show that CAR agonist CITCO inhibits growth and expansion of BTSCs in culture and xenograft model. Our findings highlight that BTSCs can be targeted through CAR for the treatment of brain tumour patients.

## Materials and methods

### Tissues, cells and reagents

The T98G and U87MG human glioma cells were obtained from American Type Culture Collection (ATCC, Manassas, VA, USA). The DB29 and DB33 human glioma cells were established in the laboratory by culturing brain tumour samples obtained from the tissue repository at Methodist Research Institute (Indianapolis, IN, USA) with an IRB approved protocol. The glioma cells were cultured in Dulbecco's modified Eagle's medium (DMEM) with 10% FBS, 1 mM sodium pyruvate, 100 U ml^−1^ penicillin G, 100 *μ*g ml^−1^ streptomycin, 2 mM glutamine, 1 mM MEM non-essential amino acids and 50 *μ*M 2*β*-mercaptoethanol in 5% CO_2_ incubator at 37°C. The glioma cells were dissociated using 0.25% trypsin with 0.05 mM EDTA solution and subcultured once in 3–5 days. Primary human astrocytes (HAs) were obtained from ScienCell (Carlsbad, CA, USA) and cultured in astrocyte medium. The human CAR agonist CITCO was obtained from BioMol (Plymouth Meeting, PA, USA). The CITCO is an imidazothiazole derivative (MW 436.7) with an EC_50_ of 49 nm and >50-fold selectivity to CAR over pregnane X receptor (PXR), and no activity on other nuclear receptors.

### Gliosphere culture

To generate gliospheres, we have adopted a culture condition as standardised in our laboratory (Chearwae and Bright, 2008). Briefly, the glioma cells cultured as monolayer in DMEM were dissociated using trypsin–EDTA and cultured in neurobasal medium (NBM) supplemented with B27 (Invitrogen, Madison, WI, USA) in the presence of 10 ng ml^−1^ EGF and bFGF. The cells were cultured in 12-well plates (5 × 10^4^ per ml per well) with different doses of CITCO in 5% CO_2_ incubator at 37°C. The gliospheres generated in primary cultures were photographed after 5–10 days using BX40 Olympus microscope (Olympus America Inc., Center Valley, PA, USA).

### Isolation of BTSCs and CD133^+^ BTSCs

To isolate BTSCs, the gliospheres were harvested after 5–7 days and dissociated by incubating (10 × 10^6^ ml^−1^) in Accutase (Sigma, St Louis, MO, USA) at 37°C for 30 min. The cells were washed and resuspended in fresh NBM, and used as BTSCs for the experiments. To isolate CD133^+^ BTSCs, the dissociated gliosphere cells (BTSCs) were resuspended (10 × 10^6^) in 200 *μ*l of PBE buffer (PBS, pH7.2, 0.5% BSA, 2 mM EDTA). Biotin conjugated anti-CD133 antibody (20 *μ*l) and FcR blocking reagent (30 *μ*l) (Miltenyi Biotech, Auburn, CA, USA) were added and incubated at 4°C for 30 min. The cells were washed and incubated in 200 *μ*l PBE buffer with 50 *μ*l streptavidin-conjugated magnetic microbeads (Miltenyi Biotech, Auburn, CA, USA) at 4°C for 30 min. The cells were passed through a MACS-LS column equilibrated with PBE buffer placed on a Midi-Macs Magnet (Myltenyi Biotech Inc., Auburn, CA, USA), and the CD133 negative cells in the flow-through were collected. The column was removed from the Magnet, and the CD133-positive cells were flushed out with the buffer using a plunger. The cells were washed and resuspended in fresh NBM for experiments.

### Immunostaining and flow cytometry

The gliospheres were cultured in fresh NBM with B27 and 10 ng ml^−1^ EGF+bFGF in the presence of different concentrations of CITCO in 12-well tissue culture plates in 5% CO_2_ incubator at 37°C. After 48 h, the gliospheres were fixed in 1% paraformaldehyde and stained with anti-Ki67 Ab (1 : 100, Santa Cruz, Santa Cruz, CA, USA) in PBS with 1% BSA at 4°C for 2 h. Alexa 488-conjugated 2nd Ab (1 : 1500, Cell Signaling, Danvers, MA, USA) diluted in PBS with 1% BSA was added for 1 h and photographed using a fluorescent microscope (DMRB, Leica, Bannockburn, IL, USA). To determine the percentage of CD133^+^ BTSCs, gliospheres cultured with different doses of CITCO for 48 h were dissociated using Accutase, followed by resuspension in PBS containing 0.1% BSA and incubated with anti-CD133 Ab (1 : 100, Cell Signaling) at 4°C for 2 h. The cells were washed and incubated with Alexa 488-conjugated secondary Ab at 4°C for 1 h, and acquired using FACS Calibur Flow Cytometer (BD Biosciences, San Jose, CA, USA) and analysed using FlowJo 8.2.6 software (Ashland, OR, USA).

### Quantitative real-time RT–PCR

Total RNA was extracted from glioma and BTSCs using RNesy kit (Qiagen, Valencia, CA, USA). Equal amount of RNA was reverse transcribed into cDNA, and quantitative RT–PCR (qRT–PCR) was performed using 384-well TaqMan Low Density Human Nuclear Receptor Array Card (384 well) in 7900 HT Fast Real time PCR system (Applied Biosystems, Foster City, CA, USA). To detect CAR transcription, 5 *μ*g total RNA was reverse transcribed into cDNA by incubating in 10 *μ*l reaction of random hexamer primers and master mix from TaqMan reverse transcription kit (Applied Biosystems, Branchburg, NJ, USA). For qRT–PCR, 2 *μ*l of the cDNA was amplified using TaqMan Universal Master Mix with optimised concentrations of CAR primer sets and probes in a standard optical 96-well reaction plate. The results were analysed using the Prism 7900 (Applied Biosystems, Carlsbad, CA, USA) relative quantification (delta delta Ct) study software (Mo *et al*, 2008). The level of CAR transcription was normalised to 18S and expressed as fold change compared with control.

### The SDS–PAGE and western blot

The glioma and BTSCs were cultured in the absence or presence of CITCO at 37°C for different time points. Whole-cell lysates were prepared by boiling in lysis buffer (0.2 M Tris-HCl, pH 6.8, 0.8 *μ*g ml^−1^ SDS, 4% glycerol, 0.59 M *β*-mercaptoethanol, 0.05 M EDTA, 8 *μ*g ml^−1^ bromophenol blue) for 5 min. Total proteins were resolved on 10% SDS–PAGE (BioRad, Hercules, CA, USA) and transferred to nylon/PVDF membrane (Millipore, Bedford, MA, USA) using Novablot transfer system (Pharmacia, Piscataway, NJ, USA). The residual binding sites in the membrane were blocked with PBST (PBS and 0.1% Tween 20) containing 5% non-fat milk powder for 1 h and incubated with anti-CAR (1 : 1000), anti-GAPDH (1 : 1000) or anti-*β*-actin (1 : 5000) antibody in PBST containing 1% milk powder at 4°C overnight. The membranes were washed in PBST, incubated with peroxidase-conjugated anti-IgG antibody (1 : 10 000) for 1 h and developed by superior signal West Pico chemiluminescence reagent (ThermoScientific, Rockford, IL, USA).

### Proliferation assay

The proliferation of glioma and BTSCs was measured by WST-1 (4-(3-(4-iodophenyl)-2-(4-nitrophenyl)-2H-5-tetrazolio)-1,3-benzene disulfonate) and ^3^H thymidine uptake assay. Briefly, the glioma cells were cultured in 96-well tissue culture plates (5000 cells per well) in DMEM without phenol red with L-glutamine, pyruvic acid, sodium salt in the presence of 10% charcoal-stripped FBS (Invitrogen) and 1% penicillin–streptomycin. The glioma cells were also cultured in 96-well tissue culture plates (1 × 10^4^ per 200 *μ*l per well) in NBM with B27 in the presence of 10 ng ml^−1^ EGF+bFGF (gliosphere). The dissociated gliosphere cells (BTSCs) and purified CD133^+^ BTSCs were cultured in 96-well tissue culture plates (1 × 10^4^ per 200 *μ*l per well) in NBM with B27 and 10 ng ml^−1^ EGF+bFGF. Increasing concentrations of CITCO were added at the initiation of cultures. The WST-1 reagent (10 *μ*l per well, Roche, Indianapolis, IN, USA) was added after 48 h, and the OD measured at 480 nm after 1–3 h using a titre plate reader. The ^3^H thymidine (0.5 *μ*Ci ml^−1^) was added at 24 h, and the cells were harvested after 48 h manually or using a Tomtech harvester 96 (Hamden, CT, USA). The amount of ^3^H thymidine uptake was counted on a Wallac Microbeta liquid scintillation counter (Perkin Elmer, Fremont, CA, USA).

### Cell cycle analysis

To determine the effect of CITCO on cell cycle progression, gliospheres were cultured in NBM with B27 and 10 ng ml^−1^ EGF+bFGF in the presence of different concentrations of CITCO. After 48 h, the cells were dissociated with Accutase and incubated in PBS containing 100 *μ*g ml^−1^ propidium iodide, 0.6% NP-40 and 20 *μ*g ml^−1^ RNase (Sigma Chemicals, St Louis, MO, USA) at 4°C for 1 h. The percentages of BTSCs at different cell cycle stages (G0/G1, G2/M and S phases) were determined based on DNA content by flow cytometry using FACS Calibur Flow Cytometer (BD Biosciences), and analysed using ModFit LT2.0 software (Verity software house, Topsham, ME, USA).

### Apoptosis assay

To determine the effect of CITCO on apoptosis, BTSCs were cultured in NBM with B27 and 10 ng ml^−1^ EGF+bFGF in the presence of CITCO. After 48 h, the cells were harvested, washed in PBS and stained with Annexin V-FITC (Roche, Indianapolis, IN, USA) in binding buffer (0.1 M Hepes/NaOH, pH 7.4, 1.4 M NaCl, 0.2 *μ*M) containing 100 *μ*g ml^−1^ propidium iodide according to the manufacturer's instruction (Roche). The cells were incubated at room temperature for 30 min in dark, acquired using FACS Calibur Flow Cytometer (BD Biosciences) and analysed using FlowJo 8.2.6 software.

### Induction, treatment and evaluation of BTSC xenograft

To determine the *in vivo* effect of CAR agonists on BTSCs, we induced BTSC xenograft in nude mice. All the animal protocols used for *in vivo* experiments were reviewed and approved by the institutional animal care and use committees at Methodist Research Institute, and performed accordingly. Six- to eight-week-old male athymic nude mice were obtained from Harlan (Indianapolis, IN, USA) and maintained under specific pathogen-free conditions in the animal care facility at Methodist Research Institute. The BTSCs were isolated by dissociating U87MG gliospheres and transplanted (25 × 10^4^) subcutaneously in 100 *μ*l NBM in the dorsum of 8-week-old nude mice. The mice were treated intraperitoneally with 25 and 100 *μ*g CITCO in 25 *μ*l DMSO on days 22, 24, 26, 30 and 36 following xenograft. The control mice received only 25 *μ*l DMSO. Tumours were measured once a week till day 50 using a digital Vernier Calipers (Marathon, Ontario, Canada), and the tumour volume (*T*_V_) was calculated as follows: *T*_V_=½(*lw*^2^) (*l*=length, *w*=width) (Euhus *et al*, 1986; Jiang *et al*, 2010; Johns *et al*, 2010). Each group contained at least four mice, and the experiments were repeated twice. On day 50, the BTSC xenografts were dissected, fixed in buffered formalin, embedded in paraffin and sliced into 6 *μ*m thick sections. The sections were stained with haematoxylin and eosin (H&E), and photographed using a phase-contrast microscope (DMRB, Leica) (Mo *et al*, 2008). The tissue sections were deparafinised, followed by incubation with 10% goat serum in PBS to block non-specific binding sites and stained overnight with anti-Ki67 Ab (1 : 100, Santa Cruz) in PBS with 1% BSA at 4°C. Alexa 488-conjugated 2nd Ab (Cell Signaling) diluted in PBS with 1% BSA was added for 1 h ,and the sections were photographed using a fluorescent microscope (DMRB, Leica).

### Statistical analysis

The data were analysed by ANOVA (Graphpad Prism 5.0, Graphpad Software, La Jolla, CA, USA), and the ^*^P<0.05, ^**^P<0.01, ^***^P<0.001 were considered significant.

## Results

### Altered expression of nuclear receptors in BTSCs

To identify novel therapeutic targets for BTSCs, we examined the expression of nuclear receptors by qRT–PCR using TaqMan Low Density Human Nuclear Receptor Array Card (384 well). We found that T98G–BTSCs express altered levels of many nuclear receptors compared with the glioma cells (Table 1). Among the 48 nuclear receptors examined, BTSCs expressed detectable levels of ERR*β* and RXR*γ* that were not detected in glioma cells. The BTSCs also showed ⩾100-fold increase in the transcription of TLX and HNF4*α,* and between 10- and 100-fold increase in PXR, ROR*α*, ROR*γ*, NORI and LRH compared with glioma cells. Moreover, 15 nuclear receptors showed 0- to 10-fold increase in BTSCs (Table 1). On the other hand, 10 genes showed 0- to 2-fold decrease, 6 genes showed 2- to 10-fold decrease and ROR*β*, SF1 and PNR showed more than 10-fold decrease in BTSCs compared with glioma. Five nuclear receptors tested were not detected either in glioma or BTSCs (Table 1). These results suggest that BTSCs express altered levels of nuclear receptors, which may serve as novel therapeutic targets for the treatment of brain tumour.

### The CITCO induces the expression of CAR in BTSCs

**Figure 1:**
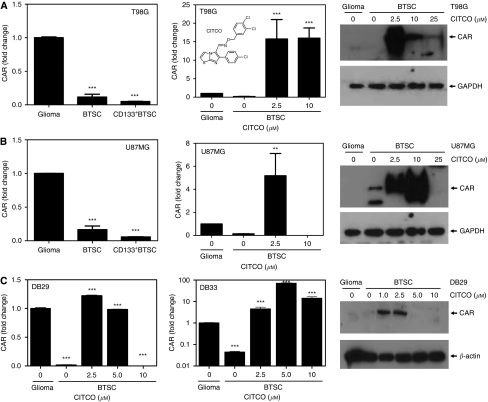
The CAR expression in BTSCs. The T98G (**A**), U87MG (**B**), DB29 and DB33 (**C**) glioma cells were cultured as monolayer in DMEM (glioma) or as gliospheres in NBM with B27 and EGF+bFGF for 5 days. The BTSCs and CD133^+^ BTSCs were purified from gliospheres and cultured in NBM with 0 to 25 *μ*M CITCO for 48 h. The CAR transcription was determined by qRT–PCR, and the data presented as fold change compared with glioma. The chemical structure of CITCO is shown as an insert (**A**). The levels of CAR protein was determined by western blot analyses. The blots were reprobed with GAPDH or *β*-actin Abs as internal controls. The figure is representative of three independent experiments. ^**^P<0.01, ^***^P<0.001.

Although many altered nuclear receptors are currently under investigation in our laboratory, in this manuscript, we present our findings on the use and mechanism of action of CAR and its agonist CITCO in targeting BTSCs for the treatment of glioma. As shown in Figure 1, qRT–PCR analyses confirmed that T98G–BTSC (A) and U87MG–BTSC (B) express significantly lower levels of CAR mRNA compared with the glioma cells. The CD133^+^ BTSCs purified from T98G (A) and U87MG (B) gliospheres showed further decrease in CAR transcription. Interestingly, treatment with 2.5 *μ*M CITCO resulted in a significant increase in the transcription of CAR in both T98G–BTSC and U87MG–BTSCs in 48 h. Western blot analyses showed that the T98G and U87MG glioma, and BTSCs express very low levels of CAR protein that increased significantly following treatment with 2.5 *μ*M CITCO in 48 h. However, higher concentrations of CITCO (⩾10 *μ*M) inhibited or abolished CAR expression in both T98G- and U87MG-derived BTSCs. Further analyses showed that the BTSCs isolated from DB29 and DB33 (C) gliospheres express lower levels of CAR compared with glioma cells, and treatment with 2.5 *μ*M CITCO resulted in significant increase in the transcription of CAR. Western blot analyses also showed that the expression of CAR protein was low in DB29–BTSCs that increased significantly after treatment with 2.5 *μ*M CITCO in 48 h (C). Higher concentrations of CITCO (⩾5 *μ*M) abolished the expression of CAR in DB29–BTSCs. These results suggest that CITCO modulates the expression of CAR in BTSCs.

### The CITCO inhibits the expansion of BTSCs

**Figure 2:**
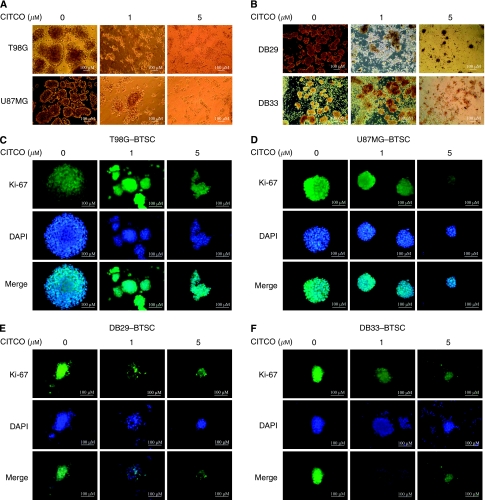
The T98G or U87MG (**A**) and DB29 or DB33 (**B**) glioma cells were cultured in NBM with B27 and EGF+bFGF in the presence of 0, 1 or 5 *μ*M CITCO. The gliospheres generated in 5–10 days were photographed (100 × ) using BX40 Olympus microscope (**A** and **B**). The T98G (**C**), U87MG (**D**), DB29 (**E**) and DB33 (**F**) gliospheres were also cultured in NBM with B27 and EGF+bFGF in the presence of 0, 1 or 5 *μ*M CITCO. After 48 h, the spheres were stained with DAPI and Ki-67 Ab, and photographed (200 × ) using a fluorescent microscope. The figures are representatives of three independent experiments.

To study the use of CAR in the regulation of BTSCs, we examined the effect of CITCO on growth and expansion in culture. Microscopic analyses showed that *in vitro* culture of T98G, U87MG, DB29 and DB33 glioma cells in NBM with B27 and EGF+bFGF resulted in the expansion of BTSCs as gliospheres in 5 days (Figures 2A and B). Interestingly, *in vitro* treatment with 1 and 5 *μ*M CITCO for 48 h induced a dose-dependent decrease in gliosphere size in all four cell types tested. Immunofluorescent microscopy showed that the gliospheres are filled with actively dividing BTSCs as evidenced by Ki-67 staining. Treatment with CITCO resulted in a dose-dependent decrease in Ki-67^+^ BTSCs in T98G (C), U87MG (D), DB29 (E) and DB33 (F) gliospheres.

**Figure 3:**
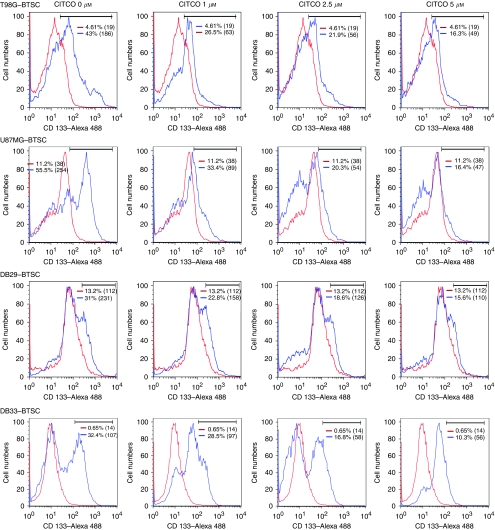
The CITCO inhibits the expansion of CD133^+^ BTSCs. The T98G, U87MG, DB29 and DB33 glioma cells were cultured in NBM with B27 and EGF+bFGF for 5 days. The gliospheres generated were further cultured in fresh NBM with 0, 1, 2.5 and 5 *μ*M CITCO for 2 days. The cells were dissociated and stained with anti-CD133 and Alexa 488-conjugated 2nd Abs, and analysed by flow cytometry. The figure shows percent CD133^+^ BTSCs (blue) and isotype control (red) with mean fluorescence intensities (MIF) in parenthesis. The figure is representative of three independent experiments.

To further determine the use of CAR in targeting BTSCs, we analysed the effect of CITCO on CD133^+^ cells by flow cytometry. We found that T98G–BTSCs displayed 43% CD133^+^ cells with a mean fluorescence intensity (MFI) of 186 that decreased dose dependently, reaching 16% with a MFI of 49 after treatment with 5 *μ*M CITCO for 48 h (Figure 3). Similarly, U87MG–BTSCs showed 56% CD133^+^ cells with a MFI of 254 that decreased to 16% with a MFI of 47 at a dose of 5 *μ*M CITCO. Further analyses showed that the DB29–BTSCs consist of 31% CD133^+^ cells with a MFI of 231 that decreased to 16% with a MFI of 110 at 5 *μ*M CITCO. Similarly, DB33–BTSCs showed 32% CD133^+^ cells with MFI of 107 that decreased to 10% with a MFI of 56 at 5 *μ*M CITCO (Figure 3). These results show that CITCO regulates the expansion of CD133^+^ BTSCs, suggesting its significance in the treatment of glioma.

### The CITCO inhibits the proliferation of BTSCs

**Figure 4:**
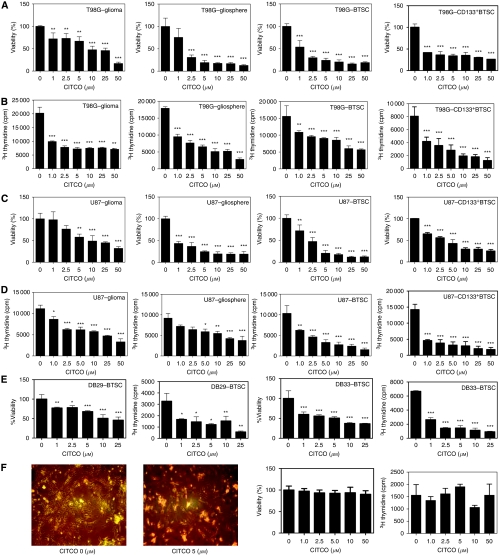
The CITCO inhibits the proliferation of BTSCs. The human glioma cells, T98G (**A** and **B**), U87MG (**C** and **D**), DB29 and DB33 (**E**), and astrocytes (**F**) were cultured as monolayers in DMEM in the absence of phenol red in the presence of 10% charcoal-stripped FBS. The glioma cells were also cultured as gliospheres in NBM with B27 and EGF+bFGF (gliosphere). The dissociated gliosphere cells (BTSC) and purified CD133^+^ gliosphere cells (CD133^+^ BTSC) were cultured in fresh NBM with B27 and EGF+bFGF. Different doses of CITCO were added at the initiation of culture. The cell viability and proliferation were measured using WST-1 and ^3^H thymidine uptake assay, respectively. The values are mean of triplicates (±s.e.m.), and ^*^P<0.05, ^**^P<0.01 and ^***^P<0.001 are considered significant. The astrocyte cultures were photographed (100 × ) under phase-contract microscope. The figure is representative of three independent experiments. ‘The color reproduction of this figure is available on the html full text version of the manuscript.’

To further test the therapeutic use of CAR in glioma, we examined the effect of CITCO on tumour cell proliferation in culture. As shown in Figure 4, we found that *in vitro* culture of T98G (A, B) and U87MG (C, D) cells in DMEM as monolayer (glioma) and in NBM (gliospheres), dissociated gliosphere cells in NBM (BTSCs) and purified CD133^+^ gliosphere cells in NBM (CD133^+^ BTSCs) resulted in a significant increase in viable cell count (A, C) and proliferation (B, D) as measured by WST-1 and ^3^H thymidine uptake assays, respectively. Interestingly, addition of CITCO resulted in a dose-dependent inhibition of viable cell count and proliferation in both T98G and U87MG glioma, and BTSCs. Similarly, *in vitro* culture of DB29- and DB33-derived CD133^+^ BTSCs in NBM resulted in a significant increase in viable cell count and proliferation that was inhibited by the addition of CITCO (E). Although CITCO induced a statistically significant inhibition at 1 and 2.5 *μ*M doses in all cell types tested, its antiproliferative effect was more pronounced in BTSCs than glioma cells. No detectable effect on the morphology, cell viability or proliferation was observed in normal HAs following *in vitro* culture with CITCO (F), suggesting its selectivity to glioma and BTSCs.

### The CITCO induces cell cycle arrest in BTSCs

**Figure 5:**
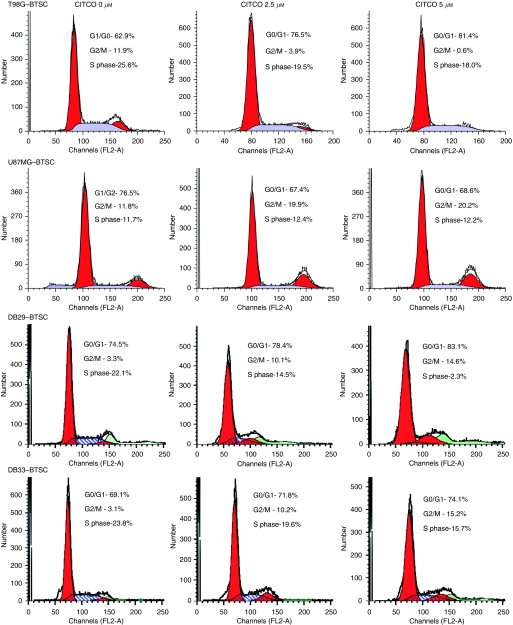
The CITCO induces cell cycle arrest in BTSCs. The T98G, U87MG, DB29 and DB33 glioma cells were cultured in NBM with B27 and EGF+bFGF. The gliospheres generated in 5 days were cultured in fresh NBM with B27 and EGF+bFGF in the presence of 0, 2.5 or 5 *μ*M CITCO. After 48 h, the gliospheres were harvested, dissociated (BTSCs), stained with propidium iodide and analysed based on DNA content by flow cytometry. The percentage of cells in G0/G1, G2/M and S phases of cell cycle are shown. The figure is representative of three independent experiments.

To explore the intrinsic mechanisms by which CAR regulates growth arrest in BTSCs, we analysed the effect of CITCO on cell cycle progression. We found that BTSCs cultured in NBM with B27 and EGF+bFGF showed distribution of cells in G0/G1, G2/M and S phases of cell cycle (Figure 5). Addition of CITCO for 24 h resulted in a dose-dependent cell cycle arrest of BTSCs. Interestingly, data analyses revealed that CITCO induces cell cycle arrest through different mechanisms in different cell types. Although CITCO increased G0/G1 cells with a decrease in G2/M and S phases in T98G–BTSCs, it decreased G0/G1 and increased G2/M without affecting the S-phase cells in U87MG–BTSCs. However, BTSCs isolated from DB29 and DB33 showed comparable responses to CITCO with increased G0/G1 and G2/M with a decrease in S-phase cells (Figure 5). These results suggest that CITCO induces cell cycle arrest differentially in different BTSCs in culture.

### The CITCO induces apoptosis in BTSCs

**Figure 6:**
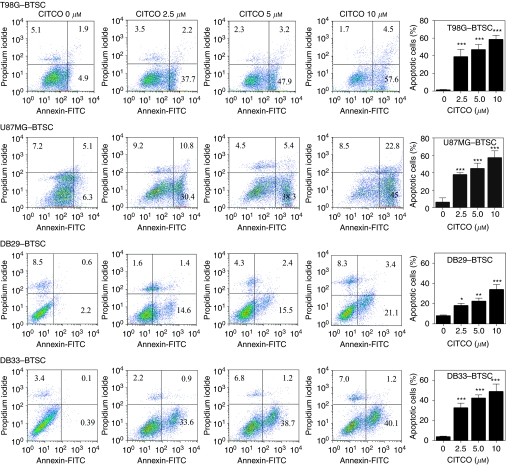
The CITCO induces apoptosis in BTSCs. The T98G, U87MG, DB29 and DB33 glioma cells were cultured in NBM with B27 and EGF+bFGF. The gliospheres generated in 5 days were cultured in fresh NBM with B27 and EGF+bFGF in the presence of 0, 2.5, 5 or 10 *μ*M CITCO. After 48 h, the gliospheres were dissociated (BTSCs), stained with Annexin V-FITC along with propidium iodide and analysed by flow cytometry. The figure is representative of three independent experiments. The histogram shows the mean Annexin V-positive BTSCs (±s.d.) of three experiments. ^*^P<0.05, ^**^P<0.001, ^***^P<0.001.

To further determine the mechanisms by which CAR regulates BTSCs, we examined the effect of CITCO on apoptosis. We found that BTSCs cultured in NBM with B27 and EGF+bFGF showed very low levels of Annexin V-positive apoptotic cells that increased dose dependently following addition of CITCO (Figure 6). The BTSCs from T98G and U87MG cultured in the absence of CITCO showed 6.8 and 11% Annexin V-positive cells that increased to 62 and 68% following addition of 10 *μ*M CITCO, respectively. Moreover, BTSCs from DB29 and DB33 gliomas showed 3 and 0.5% Annexin V-positive cells that increased to 24 and 41% following treatment with 10 *μ*M CITCO, respectively (Figure 6). These results suggest that CITCO induces apoptosis in BTSCs in culture.

### The CITCO inhibits BTSC xenograft in nude mice

**Figure 7:**
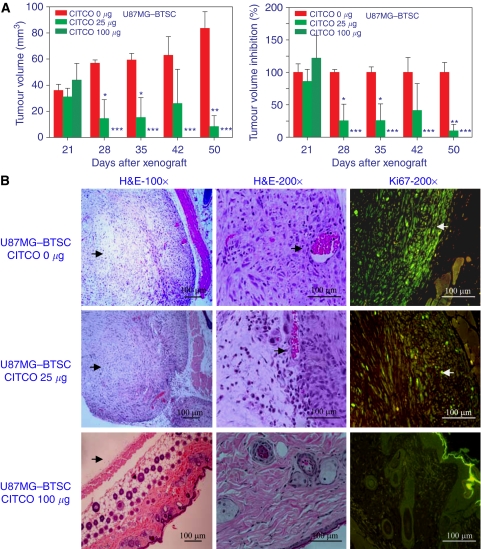
Nude mice were injected (s.c) with U87MG–BTSCs to induce tumour growth. The mice were treated (intraperitoneal) with 0, 25 or 100 *μ*g CITCO in DMSO on days 22, 24, 26, 30 and 36. (**A**) The tumour growth was measured using digital Vernier Calipers, and the tumour volume was calculated. The percent inhibition by CITCO was calculated using the mean tumour volume in the group treated with 0 *μ*M CITCO as 100%. The figure is representative of three independent experiments. (**B**) The mice were euthanised on day 50, and the BTSC xenografts were dissected; 6 *μ*m thick sections were stained with H&E or anti-Ki67 Ab and photographed (100 × /200 × ) under fluorescent microscope. ^*^P<0.05, ^**^P<0.001, ^***^P<0.001.

To determine the *in vivo* effect of CITCO on tumour growth, we used a xenograft model in nude mice. As shown in Figure 7, we found that subcutaneous transplantation of U87MG–BTSCs resulted in solid tumour growth by day 21. Interestingly, *in vivo* treatment with CITCO on days 22, 24, 26, 30 and 36 resulted in a dose-dependent decrease in tumour volume (A). In DMSO-treated control group, the tumour volume increased from 36 mm^3^ on day 21 to 84 mm^3^ on day 50, whereas in the 25 *μ*g CITCO-treated group, tumour volume decreased from 31 mm^3^ (100%) on day 21 to 8 mm^3^ (90% inhibition) on day 50. Moreover, the tumour volume in mice treated with 100 *μ*g CITCO decreased from 44 mm^3^ on day 21 to 0 mm^3^ (100% inhibition) by day 28, with no recurrence observed until day 50. Histological analysis revealed that BTSCs grow and expand as solid tumour in nude mice as evidenced by H&E staining (B). Treatment with 25 *μ*g CITCO resulted in a significant decrease in tumour growth, which further decreased to an undetectable level after treatment with 100 *μ*g CITCO (B). Furthermore, Ki-67 staining revealed that the control group showed actively dividing cells in the tumour, which decreased significantly after treatment with 25 *μ*g CITCO, with the absence of Ki-67^+^ cells at 100 *μ*g. These results suggest the *in vivo* effect of CITCO in the treatment of glioma.

## Discussion

Despite advances in modern medicine, the prognosis from current surgery, radiation and chemotherapy remains poor in brain tumour patients. The recent identification of BTSCs with resistance and recurrence properties has revolutionised the basic approaches on drug discovery and development for brain tumour. Brain tumours often present a small fraction of BTSCs, making it difficult to isolate sufficient cells for drug discovery. EGF and FGF are growth factors that promote the tumourigenicity of glioma cells (Martens *et al*, 2008). We have shown recently that EGF+bFGF induce the expansion of CD133^+^ BTSCs as gliospheres in culture (Chearwae and Bright, 2008). In this study, we show for the first time that the CAR agonist CITCO induces growth arrest and apoptosis of BTSCs in culture and in animal model.

Nuclear hormone receptors have therapeutic values in many human diseases (Blumberg and Evans, 1998). Earlier studies have demonstrated deregulated expression of nuclear receptors in glioma and their activation with specific agonists inhibited tumour growth (Berge *et al*, 2001; See *et al*, 2004). In this study, we set out to identify novel nuclear receptors that could be used to target BTSCs for the treatment of glioma. We found that BTSCs express altered levels of many nuclear receptors compared with glioma, which are currently under investigation in our laboratory. In this manuscript, we focused on determining the use of CAR agonist CITCO in targeting BTSCs. We found that the expression of CAR was low in BTSCs derived from different gliomas. The downregulation of CAR could be a mechanism by which BTSCs evade antitumour responses. Interestingly, the upregulation of CAR expression by CITCO suggests its use in targeting BTSCs in the treatment of glioma. This is consistent with our earlier report on the induction of PPAR*γ* expression by its agonists in BTSCs (Chearwae and Bright, 2008). Although the constitutively active CAR is retained in the cytoplasm, CITCO induces its translocation into the nucleus and mediates gene transcription (Baes *et al*, 1994; Kobayashi *et al*, 2003). Although the exact mechanisms are not known, our findings suggest an autoregulation of CAR expression through CITCO/CAR axis in BTSCs.

Earlier studies have shown that CAR is highly expressed in the liver and small intestine, and promotes the detoxification and elimination of potentially toxic compounds by modulating the phase I and phase II drug-metabolising enzymes (Forman *et al*, 1998; Xu *et al*, 2005; Echchgadda *et al*, 2007; Veith *et al*, 2009). Although CAR activation can disrupt thyroid hormone homoeostasis (Qatanani *et al*, 2005), it also showed protective roles in stress response (Forman *et al*, 1998; Stedman *et al*, 2005; Xu *et al*, 2005; Echchgadda *et al*, 2007). The CAR-mediated expression of xenobiotic-metabolising enzymes is generally protective, but can be deleterious, if toxic metabolites are produced (Xu *et al*, 2005). The CAR agonists induce hepatocyte proliferation that depends on c-Myc-FoxM1 function (Blanco-Bose *et al*, 2008). The CAR agonists also inhibits Fas-induced hepatocyte apoptosis, liver injury, and fatalities by depleting the proapoptotic proteins Bak (Bcl-2 antagonistic killer) and Bax (Bcl-2-associated X protein) and increasing the expression of the antiapoptotic effector myeloid cell leukaemia factor-1 (Baskin-Bey *et al*, 2006). Thus, the xenobiotic properties of CAR and its agonists have been extensively studied, but its anticancer property was not known.

In this study, we have shown that CITCO inhibits the proliferation of glioma cells in a dose-dependent manner, without affecting primary astrocytes. Interestingly, CITCO inhibits the growth and expansion of BTSCs by inducing cell cycle arrest and apoptosis in culture. Moreover, the inhibition of CD133 expression by CITCO indicates the downregulation of BTSC expansion in culture. The inhibition of solid tumour growth by CITCO in xenograft model suggests the use of CITCO in the regulation of BTSCs *in vivo*. We have shown earlier that PPAR*γ* agonists inhibit cytokine-induced activation of Jak-Stat pathway in immune cells (Natarajan and Bright, 2002) and LIF-induced activation of Jak-Stat pathway in mouse embryonic stem cells (Rajasingh and Bright, 2006). We have also demonstrated earlier that the PPAR*γ* agonists induce growth arrest and apoptosis in BTSCs by blocking EGF/FGF-induced activation of Tyk2-Stat3 pathway in BTSCs (Chearwae and Bright, 2008). Although the precise molecular basis of CAR-mediated antineoplastic effect is under investigation, our findings suggest the use of CAR agonists as a new therapy to target BTSCs for the treatment of glioma patients.

## Figures and Tables

**Table 1:** Altered expression of nuclear receptors in brain tumour stem cells

**Receptor**	**Fold change**	**Receptor**	**Fold change**
ERR*β* (NR3B2)	ND-D	TR4 (NR2C2)	0.995
RXR*γ* (NR2B3)	ND-D	TR*α* (NR1A1)	0.965
TLX (NR2E1)	628.610	ERR*α* (NR3B1)	0.934
HNF4*α* (NR2A1)	272.000	TR2 (NR2C1)	0.890
PXR (NR1I2)	27.800	RXR*α* (NR2B1)	0.848
ROR*α* (NR1F1)	19.690	EAR (NR2F6)	0.706
ROR*γ* (NR1F3)	18.280	PPAR*γ* (NR1C3)	0.648
NORI (NR4A3)	11.426	Coup TF2 (NR2F2)	0.605
LRH (NR5A2)	10.480	RAR*γ* (NR1B3)	0.565
NURR1 (NR4A2)	7.040	TR*β* (NR1A2)	0.511
NURR77 (NR4A1)	4.380	AR (NR3C4)	0.478
REVerb*α* (NR1D1)	2.960	RAR*β* (NR1B2)	0.425
MR (NR3C2)	2.507	HNF4*γ* (NR2A2)	0.353
RAR*α* (NR1B1)	2.260	Coup TF1 (NR2F1)	0.332
LXR*β* (NR1H2)	2.051	CAR (NR1I3)	0.112
PPAR*δ* (NR1C2)	1.652	FXR (NR1H4)	0.104
GR (NR3C1)	1.600	ROR*β* (NR1F2)	0.080
PPAR*α* (NR1C1)	1.421	SF1 (NR5A1)	0.0789
PGR (NR3C3)	1.330	PNR (NR2E3)	0.00023
VDR (NR1I1)	1.248	ER*α* (NR3A1)	ND-ND
RXR*β* (NR2B2)	1.195	ER*β* (NR3A2)	ND-ND
GCNF (NR6A1)	1.126	ERR*γ* (NRB3)	ND-ND
REVerb*β* (NR1D2)	1.150	DAX (NR0B1)	ND-ND
LXR*α* (NR1H3)	1.089	SHP (NR0B2)	ND-ND

Abbreviations: bFGF=basic fibroblast growth factor; BTSC=brain tumour stem cell; cDNA=complementary DNA; DMEM=Dulbecco's modified Eagle's medium; EGF=epidermal growth factor; FBS=fetal bovine serum; NBM=neurobasal medium.

The T98G glioma cells were cultured as monolayer in DMEM with 10% FBS or as gliospheres in NBM with B27 and EGF+bFGF. After 5 days, the glioma cells were harvested using trypsin+EDTA, and gliospheres were dissociated using Accutase. Total RNA was extracted from glioma and BTSCs, and reverse transcribed into cDNA using random hexamer primers and TaqMan reverse transcription kit. The gene expression profile was determined using TaqMan Universal Master Mix with nuclear receptor primer sets and probes in an optical 384-well card using the 7900 Fast Sequence Detection Real-time PCR System (Applied Biosystems). The results were analysed using the Prism 7900 relative quantification (delta delta Ct) study software. The levels of nuclear receptor gene expression are normalised to 18 s, and the values are presented as fold change in BTSCs compared with glioma cells.

## References

[bib1] Al-Hajj M, Wicha MS, Benito-Hernandez A, Morrison SJ, Clarke MF (2003) Prospective identification of tumorigenic breast cancer cells. Proc Natl Acad Sci USA 100(7): 3983–3988 12629218 10.1073/pnas.0530291100 PMC153034

[bib2] Baes M, Gulick T, Choi HS, Martinoli MG, Simha D, Moore DD (1994) A new orphan member of the nuclear hormone receptor superfamily that interacts with a subset of retinoic acid response elements. Mol Cell Biol 14(3): 1544–1552 8114692 10.1128/mcb.14.3.1544-1552.1994 PMC358513

[bib3] Bao S, Wu Q, McLendon RE, Hao Y, Shi Q, Hjelmeland AB, Dewhirst MW, Bigner DD, Rich JN (2006) Glioma stem cells promote radioresistance by preferential activation of the DNA damage response. Nature 444(7120): 756–760 17051156 10.1038/nature05236

[bib4] Baskin-Bey ES, Huang W, Ishimura N, Isomoto H, Bronk SF, Braley K, Craig RW, Moore DD, Gores GJ (2006) Constitutive androstane receptor (CAR) ligand, TCPOBOP, attenuates Fas-induced murine liver injury by altering Bcl-2 proteins. Hepatology 44(1): 252–262 16799968 10.1002/hep.21236

[bib5] Berge K, Tronstad KJ, Flindt EN, Rasmussen TH, Madsen L, Kristiansen K, Berge RK (2001) Tetradecylthioacetic acid inhibits growth of rat glioma cells *ex vivo* and *in vivo* via PPAR-dependent and PPAR-independent pathways. Carcinogenesis 22(11): 1747–1755 11698335 10.1093/carcin/22.11.1747

[bib6] Blanco-Bose WE, Murphy MJ, Ehninger A, Offner S, Dubey C, Huang W, Moore DD, Trumpp A (2008) C-Myc and its target FoxM1 are critical downstream effectors of constitutive androstane receptor (CAR) mediated direct liver hyperplasia. Hepatology 48(4): 1302–1311 18798339 10.1002/hep.22475

[bib7] Blumberg B, Evans RM (1998) Orphan nuclear receptors—new ligands and new possibilities. Genes Dev 12(20): 3149–3155 9784489 10.1101/gad.12.20.3149

[bib8] Bonnet D, Dick JE (1997) Human acute myeloid leukemia is organized as a hierarchy that originates from a primitive hematopoietic cell. Nat Med 3(7): 730–737 9212098 10.1038/nm0797-730

[bib9] Chearwae W, Bright JJ (2008) PPARgamma agonists inhibit growth and expansion of CD133+ brain tumour stem cells. Br J Cancer 99(12): 2044–2053 19018263 10.1038/sj.bjc.6604786 PMC2607234

[bib10] Dalerba P, Dylla SJ, Park IK, Liu R, Wang X, Cho RW, Hoey T, Gurney A, Huang EH, Simeone DM, Shelton AA, Parmiani G, Castelli C, Clarke MF (2007) Phenotypic characterization of human colorectal cancer stem cells. Proc Natl Acad Sci USA 104(24): 10158–10163 17548814 10.1073/pnas.0703478104 PMC1891215

[bib11] Deorah S, Lynch CF, Sibenaller ZA, Ryken TC (2006) Trends in brain cancer incidence and survival in the United States: Surveillance, Epidemiology, and End Results Program, 1973 to 2001. Neurosurg Focus 20(4): E1 10.3171/foc.2006.20.4.E1 16709014

[bib12] Echchgadda I, Song CS, Oh T, Ahmed M, De La Cruz IJ, Chatterjee B (2007) The xenobiotic-sensing nuclear receptors pregnane X receptor, constitutive androstane receptor, and orphan nuclear receptor hepatocyte nuclear factor 4alpha in the regulation of human steroid-/bile acid-sulfotransferase. Mol Endocrinol 21(9): 2099–2111 17595319 10.1210/me.2007-0002

[bib13] Euhus DM, Hudd C, LaRegina MC, Johnson FE (1986) Tumor measurement in the nude mouse. J Surg Oncol 31(4): 229–234 3724177 10.1002/jso.2930310402

[bib14] Forman BM, Tzameli I, Choi HS, Chen J, Simha D, Seol W, Evans RM, Moore DD (1998) Androstane metabolites bind to and deactivate the nuclear receptor CAR-beta. Nature 395(6702): 612–615 9783588 10.1038/26996

[bib15] Grommes C, Landreth GE, Sastre M, Beck M, Feinstein DL, Jacobs AH, Schlegel U, Heneka MT (2006) Inhibition of *in vivo* glioma growth and invasion by peroxisome proliferator-activated receptor gamma agonist treatment. Mol Pharmacol 70(5): 1524–1533 16887936 10.1124/mol.106.022194

[bib16] Jiang XD, Dai P, Wu J, Song DA, Yu JM (2010) Inhibitory effect of radiotherapy combined with weekly recombinant human endostatin on the human pulmonary adenocarcinoma A549 xenografts in nude mice. Lung Cancer10.1016/j.lungcan.2010.09.003 20965604

[bib17] Johns TG, McKay MJ, Cvrljevic AN, Gan HK, Taylor C, Xu H, Smyth FE, Scott AM (2010) MAb 806 enhances the efficacy of ionizing radiation in glioma xenografts expressing the de2-7 epidermal growth factor receptor. Int J Radiat Oncol Biol Phys 78(2): 572–578 20638193 10.1016/j.ijrobp.2010.03.027

[bib18] Kobayashi K, Sueyoshi T, Inoue K, Moore R, Negishi M (2003) Cytoplasmic accumulation of the nuclear receptor CAR by a tetratricopeptide repeat protein in HepG2 cells. Mol Pharmacol 64(5): 1069–1075 14573755 10.1124/mol.64.5.1069

[bib19] Li C, Heidt DG, Dalerba P, Burant CF, Zhang L, Adsay V, Wicha M, Clarke MF, Simeone DM (2007) Identification of pancreatic cancer stem cells. Cancer Res 67(3): 1030–1037 17283135 10.1158/0008-5472.CAN-06-2030

[bib20] Maglich JM, Parks DJ, Moore LB, Collins JL, Goodwin B, Billin AN, Stoltz CA, Kliewer SA, Lambert MH, Willson TM, Moore JT (2003) Identification of a novel human constitutive androstane receptor (CAR) agonist and its use in the identification of CAR target genes. J Biol Chem 278(19): 17277–17283 12611900 10.1074/jbc.M300138200

[bib21] Martens T, Laabs Y, Gunther HS, Kemming D, Zhu Z, Witte L, Hagel C, Westphal M, Lamszus K (2008) Inhibition of glioblastoma growth in a highly invasive nude mouse model can be achieved by targeting epidermal growth factor receptor but not vascular endothelial growth factor receptor-2. Clin Cancer Res 14(17): 5447–5458 18765536 10.1158/1078-0432.CCR-08-0147

[bib22] Mo C, Chearwae W, O’Malley JT, Adams SM, Kanakasabai S, Walline CC, Stritesky GL, Good SR, Perumal NB, Kaplan MH, Bright JJ (2008) Stat4 isoforms differentially regulate inflammation and demyelination in experimental allergic encephalomyelitis. J Immunol 181(8): 5681–5690 18832727 10.4049/jimmunol.181.8.5681 PMC2581484

[bib23] Moore LB, Parks DJ, Jones SA, Bledsoe RK, Consler TG, Stimmel JB, Goodwin B, Liddle C, Blanchard SG, Willson TM, Collins JL, Kliewer SA (2000) Orphan nuclear receptors constitutive androstane receptor and pregnane X receptor share xenobiotic and steroid ligands. J Biol Chem 275(20): 15122–15127 10748001 10.1074/jbc.M001215200

[bib24] Natarajan C, Bright JJ (2002) Peroxisome proliferator-activated receptor-gamma agonists inhibit experimental allergic encephalomyelitis by blocking IL-12 production, IL-12 signaling and Th1 differentiation. Genes Immun 3(2): 59–70 11960303 10.1038/sj.gene.6363832

[bib25] Naveilhan P, Berger F, Haddad K, Barbot N, Benabid AL, Brachet P, Wion D (1994) Induction of glioma cell death by 1,25(OH)2 vitamin D3: towards an endocrine therapy of brain tumors? J Neurosci Res 37(2): 271–277 8151734 10.1002/jnr.490370212

[bib26] O’Brien CA, Pollett A, Gallinger S, Dick JE (2007) A human colon cancer cell capable of initiating tumour growth in immunodeficient mice. Nature 445(7123): 106–110 17122772 10.1038/nature05372

[bib27] Papi A, Tatenhorst L, Terwel D, Hermes M, Kummer MP, Orlandi M, Heneka MT (2009) PPARgamma and RXRgamma ligands act synergistically as potent antineoplastic agents *in vitro* and *in vivo* glioma models. J Neurochem 109(6): 1779–1790 19457135 10.1111/j.1471-4159.2009.06111.x

[bib28] Patrawala L, Calhoun T, Schneider-Broussard R, Li H, Bhatia B, Tang S, Reilly JG, Chandra D, Zhou J, Claypool K, Coghlan L, Tang DG (2006) Highly purified CD44+ prostate cancer cells from xenograft human tumors are enriched in tumorigenic and metastatic progenitor cells. Oncogene 25(12): 1696–1708 16449977 10.1038/sj.onc.1209327

[bib29] Peacock KH, Lesser GJ (2006) Current therapeutic approaches in patients with brain metastases. Curr Treat Options Oncol 7(6): 479–489 17032560 10.1007/s11864-006-0023-8

[bib30] Qatanani M, Moore DD (2005) CAR, the continuously advancing receptor, in drug metabolism and disease. Curr Drug Metab 6(4): 329–339 16101572 10.2174/1389200054633899

[bib31] Qatanani M, Zhang J, Moore DD (2005) Role of the constitutive androstane receptor in xenobiotic-induced thyroid hormone metabolism. Endocrinology 146(3): 995–1002 15564320 10.1210/en.2004-1350

[bib32] Rajasingh J, Bright JJ (2006) 15-Deoxy-delta12,14-prostaglandin J2 regulates leukemia inhibitory factor signaling through JAK-STAT pathway in mouse embryonic stem cells. Exp Cell Res 312(13): 2538–2546 16737695 10.1016/j.yexcr.2006.04.010

[bib33] Schatton T, Murphy GF, Frank NY, Yamaura K, Waaga-Gasser AM, Gasser M, Zhan Q, Jordan S, Duncan LM, Weishaupt C, Fuhlbrigge RC, Kupper TS, Sayegh MH, Frank MH (2008) Identification of cells initiating human melanomas. Nature 451(7176): 345–349 18202660 10.1038/nature06489 PMC3660705

[bib34] See SJ, Levin VA, Yung WK, Hess KR, Groves MD (2004) 13-cis-retinoic acid in the treatment of recurrent glioblastoma multiforme. Neuro Oncol 6(3): 253–258 15279718 10.1215/S1152851703000607 PMC1871997

[bib35] Singh SK, Hawkins C, Clarke ID, Squire JA, Bayani J, Hide T, Henkelman RM, Cusimano MD, Dirks PB (2004) Identification of human brain tumour initiating cells. Nature 432(7015): 396–401 15549107 10.1038/nature03128

[bib36] Stedman CA, Liddle C, Coulter SA, Sonoda J, Alvarez JG, Moore DD, Evans RM, Downes M (2005) Nuclear receptors constitutive androstane receptor and pregnane X receptor ameliorate cholestatic liver injury. Proc Natl Acad Sci USA 102(6): 2063–2068 15684063 10.1073/pnas.0409794102 PMC548592

[bib37] Stupp R, Hegi ME (2007) Targeting brain-tumor stem cells. Nat Biotechnol 25(2): 193–194 17287755 10.1038/nbt0207-193

[bib38] Tzameli I, Pissios P, Schuetz EG, Moore DD (2000) The xenobiotic compound 1,4-bis[2-(3,5-dichloropyridyloxy)]benzene is an agonist ligand for the nuclear receptor CAR. Mol Cell Biol 20(9): 2951–2958 10757780 10.1128/mcb.20.9.2951-2958.2000 PMC85552

[bib39] Veith H, Southall N, Huang R, James T, Fayne D, Artemenko N, Shen M, Inglese J, Austin CP, Lloyd DG, Auld DS (2009) Comprehensive characterization of cytochrome P450 isozyme selectivity across chemical libraries. Nat Biotechnol 27(11): 1050–1055 19855396 10.1038/nbt.1581 PMC2783980

[bib40] Xu C, Li CY, Kong AN (2005) Induction of phase I, II and III drug metabolism/transport by xenobiotics. Arch Pharm Res 28(3): 249–268 15832810 10.1007/BF02977789

[bib41] Yang ZF, Ngai P, Ho DW, Yu WC, Ng MN, Lau CK, Li ML, Tam KH, Lam CT, Poon RT, Fan ST (2008) Identification of local and circulating cancer stem cells in human liver cancer. Hepatology 47(3): 919–928 18275073 10.1002/hep.22082

